# A Low-Rate Video Approach to Hyperspectral Imaging of Dynamic Scenes

**DOI:** 10.3390/jimaging5010006

**Published:** 2018-12-31

**Authors:** Charles M. Bachmann, Rehman S. Eon, Christopher S. Lapszynski, Gregory P. Badura, Anthony Vodacek, Matthew J. Hoffman, Donald McKeown, Robert L. Kremens, Michael Richardson, Timothy Bauch, Mark Foote

**Affiliations:** 1 Chester F. Carlson Center for Imaging Science, Rochester Institute of Technology, Rochester, NY 14623-5603, USA; 2 School of Mathematical Sciences, Rochester Institute of Technology, Rochester, NY 14623-5603, USA

**Keywords:** hyperspectral, video, imaging, coastal dynamics, moving vehicle imaging, bi-directional reflectance distribution function (BRDF), hemispherical conical reflectance factor (HCRF), stereo imaging, digital elevation model, Virginia Coast Reserve Long Term Ecological Research (VCR LTER)

## Abstract

The increased sensitivity of modern hyperspectral line-scanning systems has led to the development of imaging systems that can acquire each line of hyperspectral pixels at very high data rates (in the 200–400 Hz range). These data acquisition rates present an opportunity to acquire full hyperspectral scenes at rapid rates, enabling the use of traditional push-broom imaging systems as low-rate video hyperspectral imaging systems. This paper provides an overview of the design of an integrated system that produces low-rate video hyperspectral image sequences by merging a hyperspectral line scanner, operating in the visible and near infra-red, with a high-speed pan-tilt system and an integrated IMU-GPS that provides system pointing. The integrated unit is operated from atop a telescopic mast, which also allows imaging of the same surface area or objects from multiple view zenith directions, useful for bi-directional reflectance data acquisition and analysis. The telescopic mast platform also enables stereo hyperspectral image acquisition, and therefore, the ability to construct a digital elevation model of the surface. Imaging near the shoreline in a coastal setting, we provide an example of hyperspectral imagery time series acquired during a field experiment in July 2017 with our integrated system, which produced hyperspectral image sequences with 371 spectral bands, spatial dimensions of 1600 × 212, and 16 bits per pixel, every 0.67 s. A second example times series acquired during a rooftop experiment conducted on the Rochester Institute of Technology campus in August 2017 illustrates a second application, moving vehicle imaging, with 371 spectral bands, 16 bit dynamic range, and 1600 × 300 spatial dimensions every second.

## 1. Introduction

Hyperspectral imaging has been a powerful tool for identifying the composition of materials in scene pixels. Over the years, a large number of applications have been considered, ranging from environmental remote sensing to identification of man-made objects [1,2,3,4,5,6,7,8,9,10]. Some applications involve dynamic scenes which naturally would be well addressed by hyperspectral imaging systems operated at very high data rates. For example, coastal regions with rapidly changing conditions due to the persistent action of tides provide an example of a dynamic landscape where both the water and the land near shore change from moment to moment. Similarly, imaging of moving vehicles provides a challenging but different set of demands which would benefit from a system which can image rapidly.

The coastal zone, in particular, offers a range of important applications where hyperspectral imaging at video rates can have an impact. A wide variety of imaging systems have been used to study near-shore dynamics [11,12,13]. Considerable effort also has been made to develop hydrodynamic models that attempt to capture the dynamics of flowing sediment in the littoral zone [14], and models of flowing sediment are critical to understanding erosion and accretion processes. At the shoreline, modeling sediment transport and in particular accurately characterizing frictional effects is challenging due to the complicated dynamics as waves break on shore and then retreat [15].

Imaging of the coastal zone has taken many forms. For example, multi-spectral and hyperspectral imaging systems have been used to characterize in water constituents, bottom type, and bathymetry using radiative transfer models [7,16]. In addition, video imaging has been used to estimate flow of the water column and its constituents using video imaging (monochromatic and 3-band multi-spectral) through particle imaging velocimetry (PIV) [17,18,19]. Limitations of these past approaches are that traditional airborne and satellite remote sensing, while providing important details about sediment concentrations near shore, have produced essentially an instantaneous look at what is in fact a dynamical system. At the same time, while video systems have been used to image the water column and model its flow, the limited number of bands has meant that little information is available from these systems regarding in water constituents or bottom properties.

Relatively recently, the commercial marketplace has begun to deliver sensors which are advancing toward the long-term goal of high-frame rate hyperspectral imagery. Several different approaches have been taken, which include the use of so-called “snapshot” imaging systems [20,21]. Some hyperspectral imaging systems that fall into this category use a Fabry–Perot design [22]. However, the signal-to-noise ratio (SNR) that has been achieved by existing systems or those under development [23] is typically lower than that obtained with conventional hyperspectral imaging systems. In some cases, other trade-offs must be made to obtain comparable performance such as using fewer spectral bands or spatial pixels. Recently reported results using a snapshot imaging system based on a Fabry–Perot design indicate other issues such as mis-registration of band images during airborne data acquisition since whole band images are acquired sequentially in time [24]; this same system delivers image cubes that have 1025 × 648 spatial pixels with only 23 spectral bands and 12 bits per pixel acquired within 0.76 s, meaning that the data volumes recorded are only 9% of the data rates achieved below by our approach in comparable time (0.67 s). For our applications and the scientific goals described later in this Section, the 12-bit dynamic range found in the system described in [24] and in other snapshot hyperspectral imaging systems [25] is too limited, and this is one of the motivating factors for our having designed an overall system uses a hyperspectral line scanner with 16-bit dynamic range. On the other hand, progress has been made in co-registration of the mis-aligned band images captured via snapshot hyperspectral imaging, with one recent work demonstrating mis-registration errors of ≤0.5 pixels [26]. Some designs have included much smaller conventional hyperspectral imaging arrays that have been resampled then to a panchromatic image acquired simultaneously [27]. Of course, the potential limitation for these systems is that they may not produce hyperspectral image sequences that are truly representative of what would be recorded in an actual full-resolution imaging spectrometer.

At the same time, among conventional imaging spectrometer designs, the commercial marketplace, driven by consumer demand for portable imaging technologies as well as unmanned aerial systems (UAS) in a number of important commercial application areas, has led to cost-effective improvements to spectrographs with progressively greater sensitivity, and this in combination with improvements to data capture capabilities have together led to hyperspectral imaging systems that can frame at high rates while maintaining high data quality (low aberration) as well as excellent spectral and spatial resolution [28,29]. The current generation of conventional imaging systems requires shorter integration times, and therefore can record a line of hyperspectral pixels at much higher rates, in the 200–400 Hz range. Most of these spectrometers are incorporated in systems that operate as line scanners. The hyperspectral line-scanner incorporated in the system described in this paper is an example of a system operating with data rates in this range [30]. Line scanners such as this have been the norm in so called “push-broom” imaging system design, used in hyperspectral imaging from space- and air-borne systems [31,32,33,34,35,36,37,38,39], where the motion of the platform produces one spatial dimension, the along-track spatial dimension, of the image data cube.

In developing a system such as the the one described here in this paper, we had several specific objectives. For coastal applications, our objectives included: (1) to be able to acquire short-time-interval hyperspectral imagery time series to support long-term goals of modeling both dynamics of the near-shore water column including the mapping of in water constituents (suspended sediments, color-dissolved organic matter (CDOM), chlorophyll, etc.) and their transport, (2) to capture near-shore land characteristics (sediments and vegetation) and in particular change in sediments on short time scales due to the influence of waves and tides, (3) more broadly to be able to image from a variety of geometries from the same location in order to obtain multi-view imagery from samples of the bi-directional reflectance distribution function for use in retrieval of geophysical parameters of the surface through inversion of radiative transfer models [40] and for construction of digital surface models (DSM) to enhance derived products and contribute to validation, (4) to image at very fine-scale spatial resolutions (mm to cm in the near range) in order to derive water-column and land surface products as just described on scales where variation might occur and to consider how products derived at these resolutions then scale up to more traditional scales so often used in remote sensing from airborne and satellite platforms where resolutions have often been measured in meters, to tens of meters, or greater; and (5) to acquire imagery for all of these purposes with a hyperspectral imager with sufficient dynamic range that retrieval in both the water column and on land would be possible.

Our objectives for moving vehicle applications overlap a number of those just described for the water column, especially goals (1), (3) and (5) listed above. Our objectives here were: (a) to obtain short-time interval hyperspectral imagery, which is critical to identification and tracking of moving vehicles while minimizing distortion due to vehicle movement; (b) to be able to characterize BRDF effects for moving vehicles, and (c) to ensure that shadows due to occlusions and nearby structures in the vicinity of moving vehicles could be better characterized.

Our approach in this paper uses the very high data rates found in modern hyperspectral line scanners to achieve a low-rate hyperspectral video acquisition system. Our overall system design incorporates a modern hyperspectral imaging spectrometer integrated into a high-speed pan tilt system with onboard Inertial Measurement Unit Global Positioning System (IMU-GPS) for pointing and data time synchronization. In field settings, the system is deployed from a telescopic mast, meeting our objectives (3) and (4). By combining these components into one integrated system, we describe how a time sequence of hyperspectral images can be acquired at ∼1.5 Hz, thus operating as a low-rate hyperspectral video of dynamic scenes and satisfying objectives (1), (2), and (a). In order to meet objectives (5) and (c) above, the imaging system that we selected had 16-bit dynamic range. Traditional video systems have been used to examine coastal regions in the past, however, these have been primarily monochromatic [15] or multi-spectral imaging systems [17] with a very limited number of spectral bands (typically 3 bands); these systems provide a more limited understanding of the dynamics of the littoral zone and have been primarily used to estimate current flow vectors. Similarly, previous studies have recognized the potential of spectral information to improve persistent vehicle tracking, but most tracking studies have used panchromatic or RGB imaging due to the cost and availability of spectral imaging equipment [41,42,43,44,45,46,47].

## 2. Approach

### 2.1. Low-Rate Hyperspectral Video System

At the heart of our approach is a state-of-the-art Headwall micro High Efficiency (HE) Hyperspec [30]. This system is advertised to achieve “frame rates” of up to 250 Hz. Here the term “frame rate” refers to the rate at which a line of hyperspectral pixels can be acquired and stored in a data capture unit. Our Headwall micro HE Hyperspec E-Series is a hyperspectral line scanner with 1600 across-track spatial pixels and 371 spectral pixels, with 16-bit dynamic range. Headwall currently manufactures both visible and near infrared (VNIR) as well as short-wave infrared (SWIR) versions of the Hyperspec. This paper describes an overall system design in which a VNIR Hyperspec is the imaging unit of the system.

**Figure 1:**
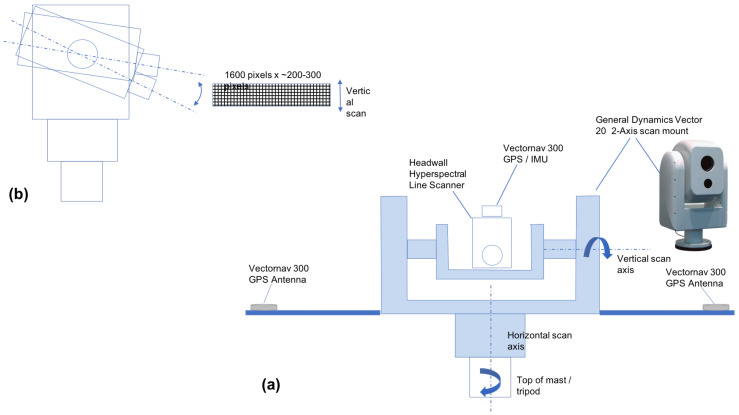
Hyperspectral video imaging concept. (**a**) Headwall Hyperspec HE E-Series hyperspectral line scanner and Vectornav 300 GPS/IMU integrated into the General Dynamics Vector 20 high-speed pan-tilt unit. (**b**) Nodding motion of the pan-tilt provides the along-track motion normally produced by movement of an aircraft when these types of imaging systems are used in an airborne platform.

Our design integrates (Figure 1) a Headwall Hyperspec into a high-speed maritime-rated General Dynamics Vector 20 pan-tilt unit [48]. Along-track motion of the Headwall Hyperspec line-scanner is accomplished by nodding of the pan-tilt unit. A Vectornav VN-300 IMU-GPS [49] is also integrated to provide pointing information for the system as well as GPS time-stamps for acquired hyperspectral data. In field settings, we mount the integrated Headwall Hyperspec and General Dynamics pan-tilt and the Headwall compact data unit atop a BlueSky AL-3 telescopic mast [50] which can raise the system from 1.5–15 m above the ground. Integration of the Hyperspec, General Dyanmics pan-tilt, and Vectornav VN-300 GPS-IMU was accomplished by Headwall under contract to RIT, and under the same contract, Headwall modified their Hyperspec data acquisition software to meet our RIT data acquisition specifications. The key data acquisition features allow direct user control of camera parameters such as integration time as well as rates of azimuthal slewing and nodding in the zenith direction of the pan-tilt system. Additional engineering, including development of custom mounting plate for the General Dynamics pan-tilt containing the Headwall Hyperspec and the Vectornav GPS-IMU components, as well as development of a field portable power supply to provide power to all components, was undertaken at RIT to further integrate the Headwall/General-Dynamics/Vectornav configuration onto the BlueSky AL-3 telescopic mast to make the final configuration field-ready.

The control software allows a variety of scan sequences to be implemented. This includes nodding at the same azimuthal orientation, typical for hyperspectral video modes, where bi-directional scanning is used to maximize hyperspectral data acquisition rates, as well as scanning sequences that step in azimuth between image frames, in combination with the normal zenith nodding mode used to produce the along track motion for each full hyperspectral image frame.

Our current instrument configuration incorporates a 12 mm lens on the Headwall Hyperspec imaging system. When operated from our telescopic mast with this lens, the Headwall system provides very fine scale hyperspectral imagery with a GSD in the millimeter to centimeter range. A table of GSD values obtainable with our system at various mast heights and distances from the mast appears in Table 1.

We note that in its current configuration, when the General Dynamics pan-tilt housing is leveled, it has a maximum deflection angle above or below the horizontal of $34^{\circ }$. This, however, is not a permanent limitation as the addition of a rotational stage in future planned upgrades will allow the system to reach and measure hyperspectral data from a much broader range of zenith angles.

### 2.2. System Calibration

To characterize the system, we used our calibration facility, which includes a LabSphere Helios (Labsphere, North Sutton, NH, USA) 0.5 m diameter integrating sphere [51] paired with a calibrated spectrometer to collect radiance data in order to derive system calibration curves, signal-to-noise ratio (SNR), and noise-equivalent spectral radiance (NESR). In the examples provided here, we show results for an integration time of 2.5 ms, which is the integration time used in the surf zone hyperspectral imagery time series example provided below.

Through various system ports, our integrating sphere is configured with three different light sources (a quartz tungsten halogen (QTH) bulb and two highly stable xenon plasma arc lamps), a VNIR spectrometer (Ocean Optics, Largo, FL, USA), and two single point broad band detectors, one silicon detector (Hamamatsu Photonics, Hamamatsu City, Japan) measuring in the visible portion of the spectrum, and an Indium Gallium Arsenide (InGaAs) detector (Teledyne Judson, Montgomeryville, PA, USA) measuring the total energy in the shortwave infrared. The external illumination sources allow the instrument to be utilized as a source capable of outputting a constant illumination across the entire 0.2 m exit port, and the radiometrically calibrated detectors are capable of measuring internal illumination conditions. For calibration purposes, the sphere operates as a source, utilizing the two high intensity plasma lamps (Labsphere, North Sutton, NH, USA), which are capable of producing almost full daylight illumination conditions through the exit port.

In our calibration, we use typically in the range of 10–30 different illumination levels, and we average 255 scans at the desired integration time. The Xenon plasma lamps provide a highly stable illumination source for the measurements, however, in order to minimize any residual instrument drift, we use two sets of dark current measurements, one before and one after imaging system measurements for the various illumination levels provided by the integrating sphere. According to manufacturer specifications each lamp maintains an approximate correlated color temperature of 5100 K ± 200 K with rated lifetime of 30,000 h. Because the Plasma External Lamps (PEL) are microwave induced sources the emitter requires feedback to maintain desired light levels. This fluctuation results in a 0.1 Hz sawtooth shaped waveform. In rest mode short term stability is ±3% from peak to peak (P-P) resulting in 6% change in magnitude of desired output. To further reduce error, Labsphere has implemented a Test Mode, during which the short term drift is ±0.5% P-P, (0.6% magnitude). Test Mode can only be maintained for a 30 min period, after which the system requires a minimum of 5 min before the next activation cycle. The long term stability reported by Labsphere for every 100 h is less than 1%. Correlated Color Temperature (CCT) change for the same time period was reported to be <100 K. Lastly observed spectral stability had fluctuations <0.5 nm for every 10 h. The quoted stability values provided here are manufacturer specifications, indicating expected performance. To obtain the results provided below, we used the Test Mode during data collection.

**Figure 2:**
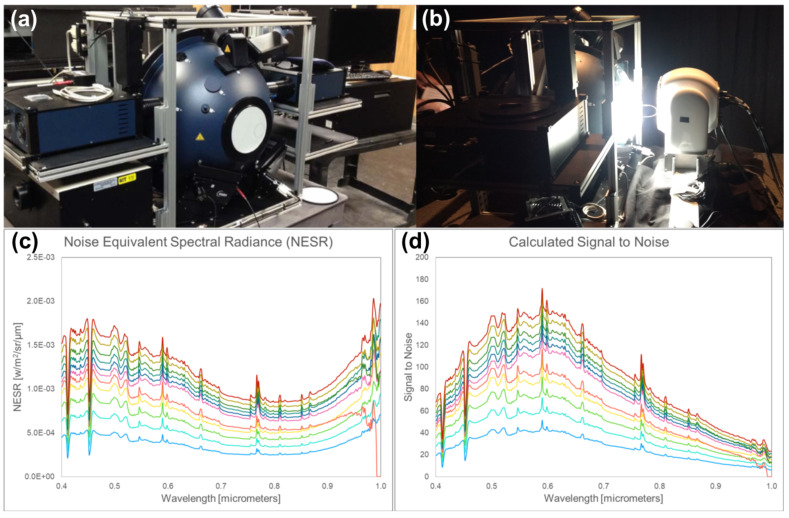
(**a**) Labsphere Helios 0.5 m diameter integrating sphere in our Rochester Institute of Technology (RIT) calibration laboratory. Plasma lamps, attached to the sphere, are visible on the top shelves to the left and right. (**b**) Our Headwall imaging system in the pan-tilt unit in front of the sphere during calibration. (**c**) typical NESR curves for 10 light levels up to the maximum output of the two plasma lamps, near daylight levels. (**d**) Typical SNR obtained over the same 30 light levels for a 2.5 × 10${}^{-3}$ s integration time. Hyperspectral video sequences shown in this paper used either a 2.5 × 10${}^{-3}$ s or 3.0 × 10${}^{-3}$ s integration time.

To develop calibrations for each wavelength, we perform a linear regression between the NIST-traceable light levels (radiance) as recorded by the onboard spectrometer attached to our integrating sphere and the recorded radiance at each wavelength in our Headwall Hyperspec imaging system. We measure system dark current by blocking the entrance aperture with the lens cap in the dark room of the calibration facility. Figure 2 shows the noise equivalent spectral radiance (NESR) and signal-to-noise ratio (SNR) for the 2.5 ms integration time used by our Headwall system during the acquisition of the hyperspectral imagery time series of the surf zone described later in this paper. The curves correspond to different illumination levels, varying from the base noise of the system to just below the saturation limit of the detector at 30,000 electrons. The SNR curves in Figure 2 show that at near full daylight levels, the peak SNR in the visible part of the spectrum is around 150, while at 0.9 μm in the near infra-red, the SNR drops to around 40. Note that the spatial resolution of our system is usually quite high (mm to cm range, as shown in Table 1, depending on the height of the mast and proximity of the ground element to the sensor). Thus, if higher SNR is desired, spatial binning by even a modest amount can provide significant enhancements; for example a 3 × 3 spatial window would provide a peak SNR of 450 at the peak in the visible and 120 at 0.9 μm.

### 2.3. Imaging the Dynamics of the Surf Zone

Imaging of the coastal zone has taken many forms. For example, multi-spectral and hyperspectral imaging systems have been used to quantify concentrations of water constituents and characterize bottom type and bathymetry using radiative transfer models [7,52]. In addition, video imaging has been used to estimate flow of the water column and its constituents using video imaging (monochromatic and 3-band multi-spectral) through particle imaging velocimetry (PIV) [18,19]. Limitations of these past approaches are that traditional airborne and satellite remote sensing, while providing important details about sediment concentrations near shore, have produced essentially an instantaneous look at what is in fact a dynamical system. At the same time, while video systems have been used to image the water column and model its flow, the limited number of bands has meant that little information is available from these systems regarding in water constituents or bottom properties.

The dynamics of sediment flow in coastal settings plays a significant role in the evolution of shorelines, determining processes such as erosion and accretion. As sea levels continue to rise, improved modeling of the evolution of coastal regions is a priority for environmental stewards, natural resource managers, urban planners, and decision makers. Understanding the details of this evolution is critical and improved knowledge of the dynamics of flowing sediment near shore can contribute significantly to hydrodynamic models that ultimately predict the future of coastal regions. Imaging systems have been used to acquire snapshots of the coastal zone from airborne and satellite platforms. Multi-spectral and especially hyperspectral imaging systems can provide an instantaneous look at the distribution of in-water constituents, bottom-type, and depth; however, these have not produced a continuous time series that looks at the short time scale dynamics of the flowing sediment. Video systems have also been used to examine coastal regions, however, these have been primarily monochromatic [15] or multi-spectral imaging systems [17] with a very limited number of spectral bands (typically 3 bands); these systems provide a more limited understanding of the dynamics of the littoral zone and have been primarily used to estimate current flow vectors without the ability to determine local particle densities. In the Results section below, we demonstrate a low-rate hyperspectral video time series. This imaging demonstration offers the advantage of bringing together the power of spectral imaging to estimate in water constituents and bottom type along with low-rate video that offers the potential to track the movement of these in-water constituents on very short time scales.

### 2.4. Real-Time Vehicle Tracking Using Hyperspectral Imagery

In recent years, vehicle detection and tracking has become important in a number of applications, including analyzing traffic flow, monitoring accidents, navigation for autonomous vehicles, and surveillance [41,47,53,54,55]. Most traffic monitoring and vehicle movement applications use relatively high-resolution video and have a high number of pixels on each vehicle, which allows tracking algorithms to rely on appearance features in the spatial domain for detection and identification. Tracking from airborne imaging platforms, on the other hand, poses several unique challenges. Airborne imaging systems typically have fewer pixels representing each vehicle within the scene due to the longer viewing distance, as well as being prone to blur or smear due to the relative motion of the sensor and the object and parallax error [41,56]. Beyond imaging system limitations, vehicle tracking/detection algorithms also must be able to handle complex, cluttered scenes that include traffic congestion and occlusions from the environment [41,47]. Occlusions are more common in airborne images and are particularly challenging for persistent vehicle tracking. When a tracked vehicle is obscured by a tree or a building, it is common for it to be assigned a new label once it reemerges. This can be avoided if the vehicle can be uniquely identified, however while traditional tracking methods can rely on high-resolution spatial features, the low resolution of airborne imagery, where the object is only represented by 100–200 or fewer pixels, makes reidentification by spatial features difficult and can lead to the tracker following a different vehicle or dropping the track entirely [41,56].

Compared to panchromatic or RGB systems, hyperspectral sensors can more effectively identify different materials based on their spectral signature and can thereby provide additional spectral features that can reidentify vehicles. Vodacek et al. [47] and Uzkent et al. [56] suggested using a multi-modal sensor design consisting of a wide field of view (FOV) panchromatic system alongside a narrow FOV hyperspectral sensor for real-time vehicle tracking and developed a tracking method leveraging the spectral information. Due to the lack of hyperspectral data, the method—along with subsequent additions to the tracking system—has only been tested on synthetic hyperspectral images generated by the Digital Imaging and Remote Sensing Image Generation model [41,56]. Results using the synthetic data have demonstrated that the spectral signatures can provide the necessary information to isolate targets of interest (TOI) in occluded backgrounds. This can be especially important when tracking vehicles in highly congested traffic or in the presence of dense buildings or trees within the scene. Experiments in cluttered synthetic scenes have shown that utilizing the spectral data outperforms other algorithms for persistent airborne tracking [56]. In addition, there has been increased recent interest in using advanced computer vision and machine learning algorithms to efficiently exploit the large amount of information contained in hyperspectral video, but no data sets currently exist with which to train—much less validate—a neural network model.

## 3. Results

### 3.1. Hyperspectral Data Collection Experiment

**Figure 3:**
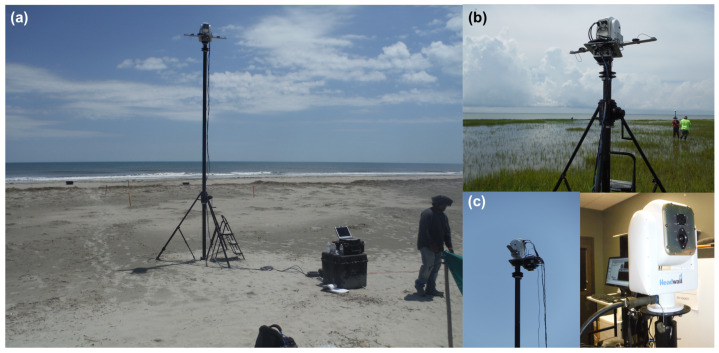
Data collection with the integrated imaging system: Headwall Hyperspec imaging system, General Dynamics maritime pan-tilt, and Vectornav-300 GPS IMU atop a BlueSky AL-3 telescopic mast. (**a**) on the western shore of Hog Island, VA while imaging littoral zone dynamics; (**b**) on the eastern shore imaging coastal wetlands; (**c**) close-ups of the imaging system while in operation at Hog Island and in the lab during testing. Closer to the shoreline in (**a**), white Spectralon calibration panels are deployed; also visible are various fiducials (orange stakes) used for image registration and geo-referencing. Fiducials were surveyed with real-time kinematic GPS.

**Figure 4:**
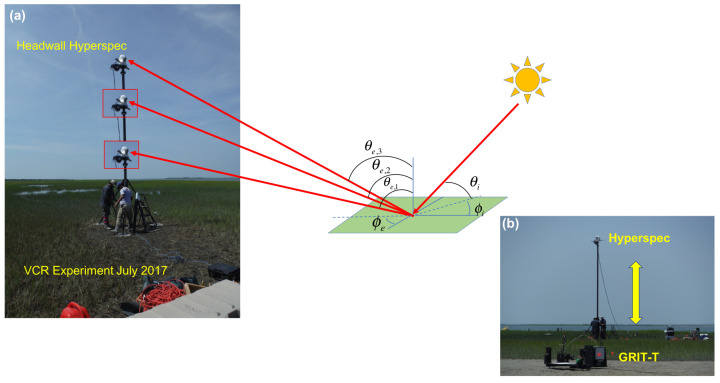
(**a**) Hyperspectral HCRF imagery sequences from our integrated hyperspectral Hyperspec imaging system atop a telescopic mast. Mast height determines view zenith angle. (**b**) The Hyperspec imaging a salt panne region during the July 2017 experiment on Hog Island while the Goniometer of the Rochester Institute of Technology-Two (GRIT) [62] records HCRF from the surface.

The first demonstration of the hyperspectral low-rate video imaging concept that we have described took place during an RIT experiment on Hog Island, VA, a barrier island which is part of the Virginia Coast Reserve (VCR) [57], a National Science Foundation Long-Term Ecological Research (LTER) site [58]. Over an 11-day period, the imaging system was used repeatedly from atop the BlueSky telescopic mast system (Figure 3) to acquire a wide variety of hyperspectral imagery of the island. By integrating the system onto the telescopic mast, the system is also able to acquire imagery from the same region on the ground, or of the water, from multiple viewing geometries, allowing the bi-directional reflectance distribution function (BRDF) of the surface to be sampled in collected imagery (Figure 4). For field data collections such as these, the term hemispherical conical reflectance factor (HCRF) is also sometimes used as a descriptor since: (a) the sediment radiance is compared with the radiance of a Lambertian standard reference (Spectralon panel), (b) the sensor has a finite aperture, and (c) the primary illumination source is not a single point source but contains both direct (solar) and indirect sources of illumination (skylight and adjacency effects) [59,60,61]. In our experiment, described in greater detail below, we deployed our hyperspectral field-portable goniometer system, the Goniometer of the Rochester Institute of Technology-Two (GRIT-T) [62] for direct comparison with hyperspectral imagery acquired from our Headwall integrated hyperspectral imaging system at varying heights on the telescopic mast. HCRF of the surface provides information on the geophysical state of the surface, such as the fill factor, which can be inferred by inverting radiative transfer models [40], which has been done previously using GRIT-T hyperspectral multi-angular data [63].

### 3.2. Digital Elevation Model and HCRF from Hyperspectral Stereo Imagery

**Figure 5:**
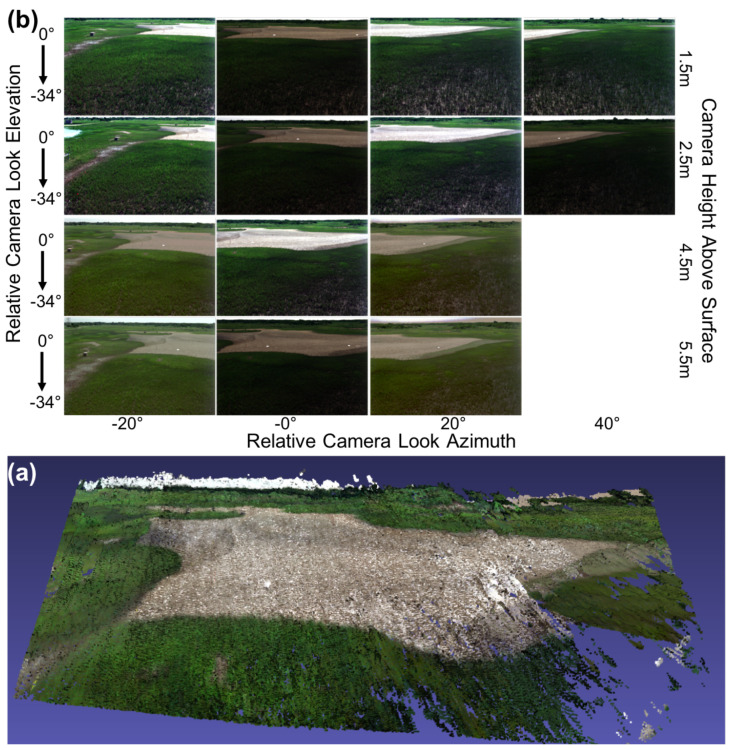
(**a**) DEM-derived from multi-view imagery from our mast-mounted hyperspectral system. (**b**) the fourteen hyperspectral scenes used as input to a Structure-from-Motion (SFM) algorithm. These hyperspectral scenes had spatial dimensions 1600 × 971 with 371 spectral bands.

The hyperspectral imagery acquired from differing viewing geometries of the same surface also allows us to develop digital elevation models (DEMs) of the surface which can be merged with the hyperspectral imagery and used in modeling and retrieval of surface properties. An example DEM-derived from the multi-view hyperspectral imagery that we acquired at our study site on Hog Island in July 2017 appears in Figure 5, which shows the resulting DEM from a set of fourteen hyperspectral scenes acquired from our mast-mounted system. DEM construction used the structure from motion (SFM) algorithm PhotoScan developed by AgiSoft LLC [64]. Similar results have been obtained from stereo views of a surface from unmanned aerial system (UAS) platforms [38] as well as from a ground-based hyperspectral imaging system [65], although the latter result was obtained from significantly longer distances, requiring the use of atmospheric correction algorithms. The example provided in Figure 5 was for stand-off distances significantly less than those for which atmospheric correction would be necessary.

Each image of the set of 14 used in creating the DEM is a hyperspectral scene acquired with the full 371 spectral bands from 0.4–1.0 μm, 1600 across track spatial pixels, and 971 spatial pixels in the second spatial dimension produced by the nodding of the pan-tilt. Each row was produced from a series of scans that overlapped in azimuth and were acquired at different mast heights. In these examples, the height of the hyperspectral imager above the surface during image acquisition was respectively 1.5 m, 2.5 m, 4.5m, and 5.5 m. Each scene shows a salt panne surrounded by coastal salt marsh vegetation, predominantly *Spartina alterniflora*.

Having the ability to produce a DEM as part of the data collection workflow has potential advantages. Lorenz et al. [65] used this information to correct for variations in illumination over rocky outcrops by determining the true angle of the sun to the surface normal derived from the DEM. For our own workflow, which is focused on problems such as inversion of radiative transfer models to retrieve geophysical properties of the surface [40,63], we require both the true viewing zenith and azimuth angles of our imaging system in the reference frame of the tilted surface normal as well as the incident zenith and azimuth angles of solar illumination within this tilted coordinate system. The onboard GPS-IMU of our mast-mounted imaging system, provides pointing (view orientation) and timestamps which together with the DEM allow the calculation of these angles. Fiducials placed in the scene enhance the overall accuracy of these angle calculations.

**Figure 6:**
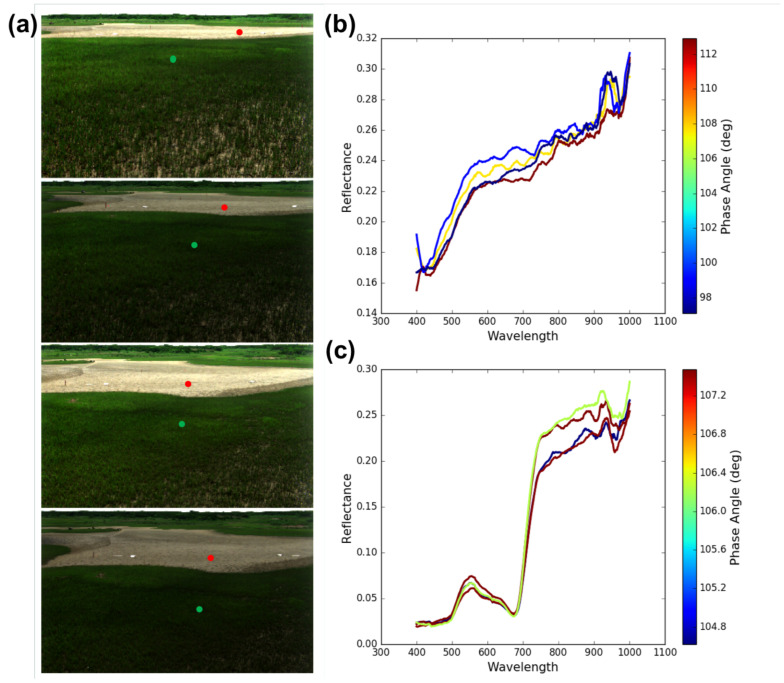
(**a**) Enlargement of four hyperspectral scenes acquired from four mast heights in the set of fourteen shown in Figure 5. (**b**,**c**) Set of spectra from the same location in each of the four scenes for (**b**) a position (red dot) in the salt panne, and (**c**) a position (green dot) in the salt marsh vegetation.

The fourteen scenes portrayed in Figure 5 also represent another important aspect of our overall approach described earlier in Section 3.1: the acquisition of multi-view imagery that sub-sample the HCRF distributions that form the core of inversion of radiative transfer models to retrieve geophysical properties of the surface [40,63] and satisfy goal (3) stated in the Introduction. Figure 6 shows examples of the spectral reflectance derived from 4 of the 14 scenes acquired from the salt panne at different mast heights, which as Figure 4 illustrates, provide us with a sub-sample of the HCRF. We have previously demonstrated an approach to retrieving sediment fill factor from laboratory bi-conical reflectance factor (BCRF) measurements [63] and then extended this to retrieval from multi-view hyperspectral time-series imagery acquired by NASA G-LiHT and multi-spectral time series imagery from GOES-R [40]; in each case, these retrievals represented a more restricted sub-sample of points from the HCRF distribution. Imagery from our mast-mounted system can allow us to more completely validate the inversion of this modified radiative transfer model to retrieve and map sediment fill factor from imagery and in particular help in assessing how many and which views of the surface are most critical for successful inversion.

### 3.3. Low-Rate Hyperspectral Video Image Sequence of the Surf Zone

**Figure 7:**
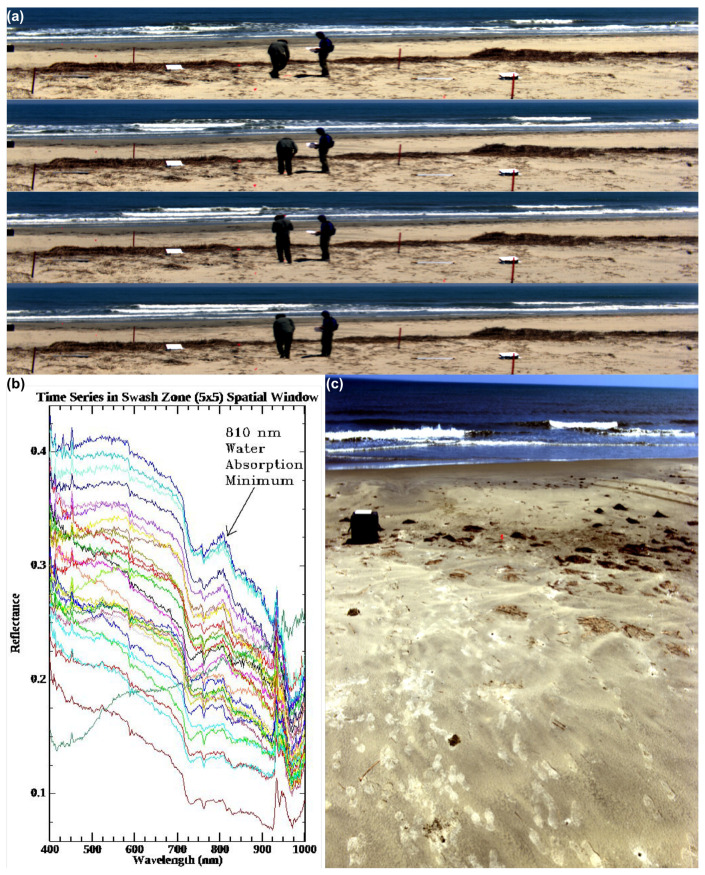
(**a**) Hyperspectral video image sequence using our integrated Headwall micro-HE VNIR hyperspectral imaging system on Hog Island, VA on 14 July 2017. The representative sequence subset (from a time series of 30 images) shown here contains hyperspectral image frames with spatial dimensions 1600 × 212 each with 371 spectral bands. Each hyperspectral scene was acquired approximately once every 0.67 s. Two Spectralon reference panels used in reflectance calculations and several orange fiducial stakes used in geo-referencing are also visible. (**b**) Spectral reflectance captured by the integrated system for a co-registered pixel in the swash zone of the hyperspectral video image sequence. The spectral reflectance, for a 5 × 5 spatial window, is shown over all 30 hyperspectral images, which were acquired once every 0.67 s. The 810 nm peak indicated corresponds to a well-known minimum in the water absorption spectrum [66], well-correlated with shallow water bathymetry [67]. (**c**) Slow scan/longer integration time hyperspectral scene with 1600 × 2111 spatial pixels and 371 spectral channels acquired closer to the waterline with the system deployed at 1.5 m height, showing the mm- to cm-scale resolution possible: the details of footprints can be clearly seen.

On 14 July 2017, our integrated system was used for the first time in the low-rate video mode to acquire imagery of the surf zone on the eastern shore of Hog Island, VA. One such image sequence is shown in Figure 7, which shows a subset of a longer sequence of images acquired every 0.67 s. Each image in the scene is 1600 across-track pixels (horizontal dimension) with 371 spectral bands each by 212 along-track pixels (vertical dimension produced by the nodding motion of the General Dynamics pan-tilt unit). The integration time for each line of 1600 across-track spatial pixels with 371 spectral pixels each was approximately 2.5 ms. Once other latencies in data acquisition are accounted for and the necessary time is allowed for the pan-tilt to reverse direction, the acquisition rate of 0.67 s for the full hyperspectral scene is achieved. We emphasize that there is no specific limitation of the system that prevents longer integration times and/or slower slewing rates from being used, and other data collected during the experiment did use slower scan rates to obtain larger scenes (see for example the 14 hyperspectral scenes in Figure 5 and the long scan in the lower right portion of Figure 7 which shows a scene with 1600 × 2111 spatial pixels and 371 spectral bands acquired over a 12-s interval during a very slow scan with a longer integration time). The latter hyperspectral scene, in particular, represents our goal (4) stated in the Introduction of being able to produce mm- to cm-scale imagery in the near range to better characterize the land surface at scales typical of the variation found near the waterline. However, in the hyperspectral low-rate video mode, image frames of the size shown in Figure 7 are typical. The quality of the spectra obtained in the imagery is indicated by the spectral time series derived from a small 5 × 5 window near the shoreline over time. A well-known local minimum in the liquid water absorption spectrum [66] normally appears in very shallow waters as a peak in the reflectance spectrum around 810 nm. This peak is well correlated with shallow water bathymetry typically in depths that are ≤1 m, and this feature was previously used in a shallow water bathymetry retrieval algorithm and demonstration which compared favorably with bathymetry directly measured in situ [67]. Obtaining spectral data of sufficient quality is important to the success of retrievals based on spectral features, such as the 810 nm feature just described, band combinations and regressions based on band combinations [68,69], “semi-analytical” models [70,71], or inversion of forward-modeled look-up tables generated from radiative transfer models such as Hydrolight [7], which rely on the spectral and radiometric accuracy of the hyperspectral data. These short-time-scale hyperspectral imagery sequences satisfy our stated goals (1) and (2).

### 3.4. Time Series Hyperspectral Imagery of Moving Vehicles

**Figure 8:**
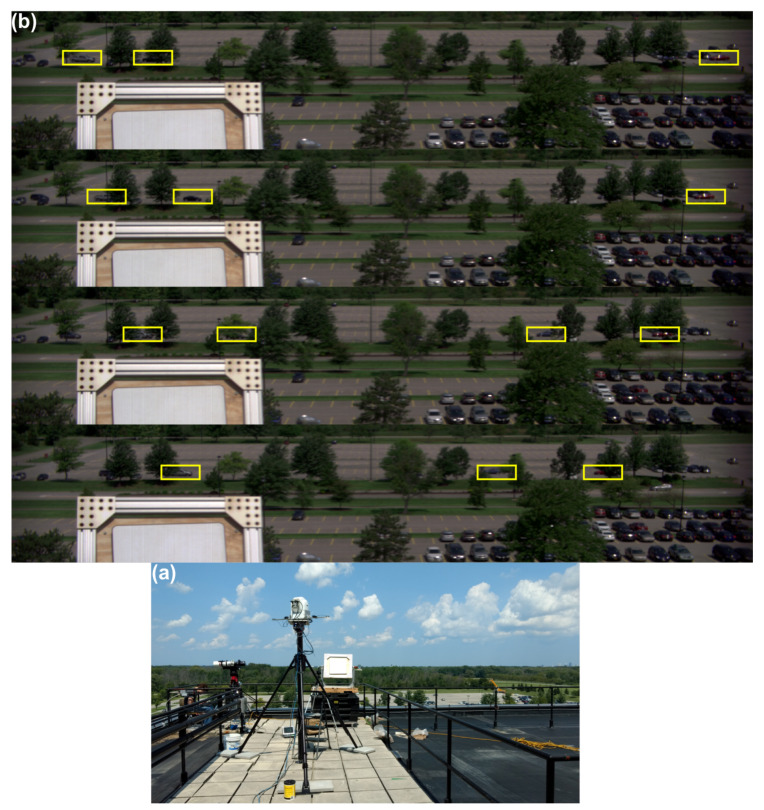
(**a**) Our integrated system deployed on the roof of the Chester F. Carlson Center for Imaging Science on the RIT campus on 9 August 2017 during a second test focused on imaging of moving vehicles. Shown also is a Spectralon panel deployed on the roof and elevated to be within the field of view of the imaging system. (**b**) Hyperspectral image time series (**top to bottom**) of the RIT parking lot showing five moving vehicles in a cluttered environment. The yellow boxes outline the positions of the test vehicles over time, but a box is drawn only when a test vehicle is clearly visible.

A second test of the video capabilities of our hyperspectral imaging system was performed on 9 August 2017 at RIT. For this experiment, imaging of moving vehicles was the primary focus. The same instrument configuration was used from atop the Chester F. Carlson Center for Imaging Science at RIT (Figure 8). Slewing rates of the pan-tilt as well as integration time of the Headwall micro HE were adjusted to achieve a larger image in the along-track dimension (zenith or nodding dimension). The integration time for this data collection was 3.0 ms, and the images in the sequence were acquired once every second. These images have 1600 across-track pixels and 299 along-track pixels with 371 spectral bands.

The objective of the data collection was to obtain hyperspectral image sequences that would be useful for studies of the detection and tracking of vehicles driving through the parking lot at ∼2.75 mps and passing behind various occlusions within the scene, such as the trees in the background and parked cars. An example sequence of hyperspectral image frames from the experiment appear in Figure 8. Each image contains the calibration panel. Note the partly cloudy conditions leading to potential rapid changes to the illumination state. Yellow boxes are drawn around the test vehicles controlled for the experiment. Note that vehicles are occluded at times so the number of boxes drawn can change from image to image. The figure illustrates the complexity of vehicle tracking when a large number of occlusions are present. Video sequences such as this will be produced in future experiments but with a longer duration and with coincident intensive reference data collection to serve as community resources. Such data sequences of short-time interval data of moving vehicles are especially useful for modeling purposes to address the challenge of occlusions (our objective (c) stated in the Introduction) and mixtures that appear in the spectral imagery. Similarly, as vehicles move through the scene, the imaging geometry changes significantly leading to BRDF effects that must be properly modeled for successful extraction and tracking of moving vehicles. The imagery shown, therefore, is important to be able to meet objectives (a) and (b) described in the Introduction.

## 4. Conclusions

We have described an approach to acquiring full hyperspectral data cubes at low video rates. Our approach integrated a state-of-the-art hyperspectral line scanner capable of high data acquisition rates into a high speed maritime pan-tilt unit. The system also included an integrated GPS/IMU to provide position and pointing information. The entire system is integrated onto a telescopic mast system that allows us to acquire hyperspectral time series imagery from multiple vantage points. This feature also makes possible the creation of a DEM from the resulting stereo hyperspectral views, an approach which was illustrated in this study. Similarly, the multi-view capability also allows the system to sample the bi-directional reflectance distribution function. We provided two examples of the low-rate hyperspectral video approach, showing hyperspectral imagery time series acquired in two different settings for very different applications: imaging of the dynamics of the surf zone in a coastal setting and moving vehicle imaging in the presence of many occlusions. We evaluated SNR and NESR and found values within acceptable limits for the data rates and integration times used in the examples. We noted that SNR could be further improved by spatial binning, an acceptable trade-off in some applications given the very high spatial resolution that we obtain with this system. Within the present system architecture, we noted that further improvements in hyperspectral image acquisition rates could be achieved by reducing the size of the across-track spatial dimension. Our particular system does not allow spectral binning on-chip, however, such capabilities do exist in commercially available systems and could be used to further accelerate image acquisition rates.

## Figures and Tables

**Table 1:** GSD (m).

Height of Camera (m)	1	2	3	4	5	6	7	8	9	10	15
**Distance from Mast (m)**											
**1**	0.0008	0.0012	0.0017	0.0022	0.0028	0.0033	0.0038	0.0044	0.0049	0.0054	0.0081
**2**	0.0012	0.0015	0.0020	0.0024	0.0029	0.0034	0.0039	0.0045	0.0050	0.0055	0.0082
**5**	0.0028	0.0029	0.0032	0.0035	0.0038	0.0042	0.0047	0.0051	0.0056	0.0061	0.0086
**10**	0.0054	0.0055	0.0057	0.0058	0.0061	0.0063	0.0066	0.0069	0.0073	0.0077	0.0098
**15**	0.0081	0.0082	0.0083	0.0084	0.0086	0.0088	0.0090	0.0092	0.0095	0.0098	0.0115
**20**	0.0109	0.0109	0.0110	0.0111	0.0112	0.0113	0.0115	0.0117	0.0119	0.0121	0.0136
**25**	0.0136	0.0136	0.0136	0.0137	0.0138	0.0139	0.0141	0.0142	0.0144	0.0146	0.0158
**30**	0.0163	0.0163	0.0163	0.0164	0.0165	0.0166	0.0167	0.0168	0.0170	0.0171	0.0182
**35**	0.0190	0.0190	0.0190	0.0191	0.0192	0.0192	0.0193	0.0195	0.0196	0.0197	0.0206
**40**	0.0217	0.0217	0.0217	0.0218	0.0218	0.0219	0.0220	0.0221	0.0222	0.0223	0.0232
**45**	0.0244	0.0244	0.0244	0.0245	0.0245	0.0246	0.0247	0.0248	0.0249	0.0250	0.0257
**50**	0.0271	0.0271	0.0271	0.0272	0.0272	0.0273	0.0274	0.0274	0.0275	0.0276	0.0283
**55**	0.0298	0.0298	0.0299	0.0299	0.0299	0.0300	0.0301	0.0301	0.0302	0.0303	0.0309
**60**	0.0325	0.0325	0.0326	0.0326	0.0326	0.0327	0.0327	0.0328	0.0329	0.0330	0.0335
